# Trimester-specific reference intervals for serum N-acetyl-β-D-glucosaminidase in healthy pregnant women in Hainan, China

**DOI:** 10.1016/j.eurox.2026.100444

**Published:** 2026-01-23

**Authors:** Fen Zhou, Yichuan Wang, Ying Zheng, Desheng Wang, Meng Chang, Shichuan Wang

**Affiliations:** Department of Clinical Laboratory, Center for Laboratory Medicine, Hainan Women and Children's Medical Center, Haikou, Hainan 570206, China

**Keywords:** N-acetyl-β-D-glucosaminidase, Reference interval, Pregnancy, Gestational age, Hainan

## Abstract

The absence of gestational age-specific reference intervals for serum N-acetyl-β-D-glucosaminidase (NAG) in pregnant women may lead to clinical misinterpretation. This study aimed to establish trimester-specific reference intervals for serum NAG in healthy pregnant women from Hainan, China, and to characterize its dynamic changes throughout gestation. In this cross-sectional study, 2416 healthy women with singleton pregnancies were stratified by gestational age into three groups: first trimester (1–12 +6 weeks; *n* = 1295), second trimester (13–27 +6 weeks; *n* = 670), and third trimester (28–40 weeks; *n* = 451). Serum NAG levels were measured, and trimester-specific reference intervals were established using the 2.5th to 97.5th percentiles. Serum NAG concentrations increased significantly with advancing gestation (*P* < 0.0001). The established reference intervals were 12.0–40.0 U/L for the first trimester, 16.0–63.2 U/L for the second trimester, and 29.3–107.0 U/L for the third trimester—all substantially higher than those of the non-pregnant control group (8.0–23.4 U/L). The median NAG level in the third trimester (56.2 U/L) represented a 143 % increase compared to the first trimester (22.5 U/L). This study provides the first gestational age-specific reference intervals for serum NAG in pregnant women in a tropical region of China. The findings confirm that physiological NAG levels increase progressively with gestational age. The use of non-pregnant reference intervals in clinical practice may lead to misclassification of renal function during pregnancy, underscoring the necessity of adopting trimester-specific reference standards in prenatal laboratory settings.

## Highlights

• The study established gestational age-stratified reference intervals for serum NAG levels.
• Serum NAG levels exhibit a progressive physiological increase throughout gestation.
• Highlighting the need for trimester-specific standards in prenatal laboratory practice.

## Introduction

Serum **N-acetyl-β-D-glucosaminidase (NAG)** is a lysosomal acid hydrolase extensively involved in cellular metabolism. It plays a critical role in the catabolism of glycoproteins by catalyzing the hydrolysis of terminal β-linked N-acetylglucosamine residues from glycoprotein substrates [1]. Due to its relatively high molecular weight (>70 kDa), NAG is not filtered through the glomerular basement membrane and therefore is typically absent from the urine under physiological conditions [2], [3]. Elevated urinary NAG levels have been widely recognized as a sensitive and early biomarker of renal tubular injury, particularly in non-pregnant populations [4], [5], [6], [7].

Emerging evidence highlights a dual physiological role of NAG during pregnancy. Serum NAG in pregnant women was related to the formation and maturation of placenta. NAG is abundantly expressed in placental decidual and chorionic tissues [8], [9], where it contributes to the degradation of glycosaminoglycans within the placental stroma—processes [10]. It was reported that NAG was parallel to the development of the placenta and the histological changes of the placental connective tissue after 4 months of gestation [11], especially in isoenzyme A form [12]. These findings suggest that NAG may serve not only as a marker of renal tubular function but also as a potential indicator of placental metabolic activity and health. Therefore, monitoring serum NAG levels during pregnancy may offer clinical value in evaluating both maternal renal status and placental function.

Pregnancy induces a series of physiological and biochemical adaptations in the mother to accommodate fetal development and maternal homeostasis. These adaptations often alter the concentration and activity of various serum biomarkers, including enzymes involved in renal function [13], [14], [15]. In this context, the enzymatic kinetics of renal tubular markers such as NAG may be affected, resulting in gestational age-specific fluctuations. Importantly, reference intervals for NAG derived from non-pregnant populations may not be applicable to pregnant women. Prior studies have demonstrated significantly elevated serum and urinary NAG activity in healthy pregnant women compared to non-pregnant controls, suggesting the necessity for pregnancy-specific reference values [16], [17].

Geographic and environmental factors may further influence biomarker dynamics. In tropical regions such as Hainan Province, high ambient temperatures and humidity levels can exacerbate physiological renal hyperfiltration during pregnancy, potentially amplifying fluctuations in NAG levels. However, no previous study has systematically established gestational age-specific or region-specific reference intervals for serum NAG in pregnant women residing in such environments.

In this study, we conducted a large-scale cross-sectional study involving 2416 healthy singleton pregnant women receiving prenatal care at the Hainan Women and Children’s Medical Center between January and December 2023. This study aimed to establish the first trimester-specific reference intervals for serum NAG in a tropical Chinese population, thereby providing a reliable clinical framework for the accurate evaluation of renal function and placental health in pregnant women under region-specific physiological conditions.

## Materials and methods

This study enrolled healthy pregnant women who attended routine prenatal examinations at the Hainan Women and Children’s Medical Center from January to December 2023. A control group comprised healthy non-pregnant women undergoing routine health check-ups at the same medical center during the same period. Pregnant women were eligible for inclusion if they had confirmed singleton intrauterine pregnancies and had complete medical documentation clearly indicating gestational age. Participants were excluded if they reported medication usage other than standard prenatal supplements (folic acid, vitamins, or calcium) during pregnancy or had a known medical history of hypertension, diabetes mellitus, liver disease, kidney disease, or pregnancy-related complications such as preeclampsia, gestational hypertension, or gestational diabetes. Individuals with a history of infectious diseases, habitual alcohol consumption, smoking, or drug abuse were also excluded from the study cohort.

All participants in the pregnant cohort were spontaneously conceived. No individuals who had undergone assisted reproductive technologies (e.g., in vitro fertilization) were included in the study cohort. Gestational age for all participants was confirmed by first-trimester ultrasound measurement of the crown-rump length (CRL). Participants in the pregnant cohort were classified into three groups based on gestational age, consistent with previously established criteria [18]: the first trimester (≤13 +6 weeks), second trimester (14–27 +6 weeks), and third trimester (≥28 weeks). Following rigorous screening, the final pregnant cohort consisted of 2416 healthy women, including 1295 women in the first trimester, 670 in the second trimester, and 451 in the third trimester. The control group consisted of 551 healthy non-pregnant women. Venous blood samples (2 mL) were collected from all participants under fasting conditions into biochemical collection tubes. Immediately following collection, samples were centrifuged at 4000 rpm for 10 min. Serum specimens demonstrating hemolysis or lipemia upon visual inspection were excluded from subsequent analyses to ensure specimen quality and reliability of the assay results. Serum NAG concentrations were determined using an automated biochemical analyzer (Mindray BS-2000; Mindray Medical International Ltd., Shenzhen, China) based on the 6-methyl-2-mercaptopyridine (MPT) substrate method. All procedures were performed in accordance with standardized operational protocols. Assay calibration was routinely performed using matched calibrators provided by the manufacturer, and internal quality controls were consistently maintained within acceptable limits throughout the study duration.

All serum samples used in this study had been collected previously as part of routine clinical care. No additional venipuncture or sample collection was performed for research purposes.

## Statistics

All statistical analyses were conducted using SPSS version 25.0. The normality of data distribution was assessed using the Kolmogorov–Smirnov (K-S) test. Reference intervals were calculated non-parametrically and expressed as the 2.5th to 97.5th percentiles [19]. Group comparisons were performed using the Kruskal-Wallis H test, with a significance threshold of p < 0.05. Post hoc pairwise comparisons were conducted using the Bonferroni correction(α=0.05/6 =0.0083), with p < 0.0083 considered statistically significant. Spearman correlation analysis was used to evaluate the relationship between NAG levels and gestational age, with p < 0.05 indicating statistical significance.

## Results

### Baseline characteristics of study population

**Fig. 1:**
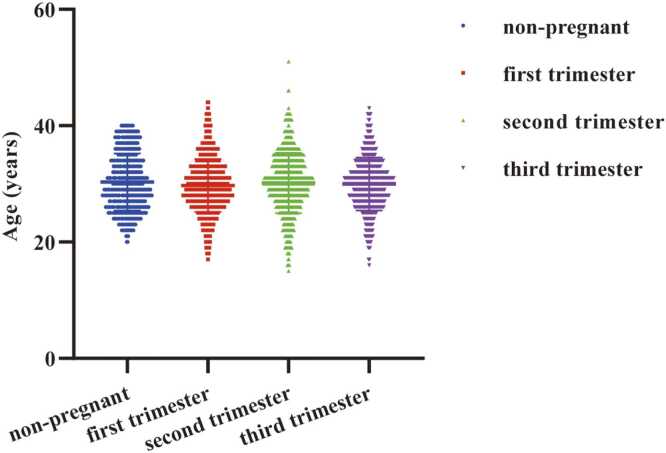
Age distribution chart of non-pregnant women and different pregnancies. first trimester (1–12^+6^ weeks), second trimester (13–27^+6^ weeks), and third trimester (28–40 weeks). When *p* < 0.05 is expressed as *, *p* < 0.01 is expressed as **, *p* < 0.001 is expressed as ***, *p* < 0.0001 is expressed as ****.

A total of 2416 healthy pregnant women and 551 healthy non-pregnant controls were included in the analysis. As summarized in Table 1, participants in the non-pregnant control group had a mean age of 30.32 ± 4.88 years. Among the pregnant women, the mean ages were 29.74 ± 4.54 years in the first trimester group (n = 1295), 30.15 ± 5.17 years in the second trimester group (n = 670), and 29.96 ± 4.56 years in the third trimester group (n = 451). No statistically significant differences in mean age were observed among these groups (Fig. 1).

**Table 1:** Demographic characteristics of non-pregnant women and women at different stages of pregnancy.

	Non-pregnant group	First-trimester group	Second-trimester group	Third-trimester group
Gestational age (weeks)	N	1–12^+ 6^	13–27^+6^	28–40
Number of cases (n)	551	1295	670	451
Age range (years)	20–40	17–44	15–51	16–43
Age (years)^#^	30.32	29.74	30.15	29.96
Hemoglobin (g/L)^#^	129.5	123.5	115.9	112.5
Creatinine (umol/L)^#^	55.48	43.22	41.43	43.86
Urea (mmol/L)^#^	3.981	2.489	2.435	2.548
Uric acid (umol/L)^#^	275.9	207.1	216.2	254.5

Note: N, not applicable; #, mean.

### Correlation analysis between serum NAG levels and maternal clinical parameters

**Fig. 2:**
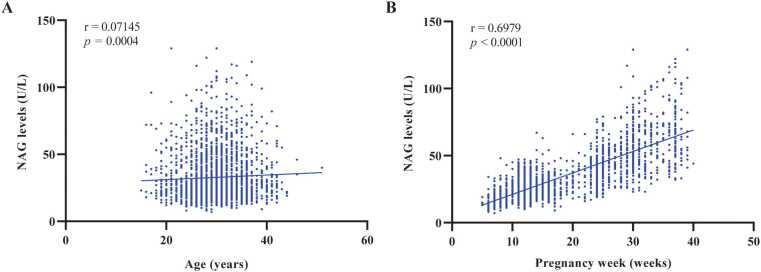
Correlation of maternal serum nag levels with age and gestational age. A. Relationship between maternal age and NAG levels. B. Relationship between gestational age and NAG levels. Abbreviations: N-Acetyl-β-D-Glucosaminidase (NAG).

Correlations between serum NAG concentrations and maternal age or gestational age were evaluated using Spearman’s correlation analysis. Serum NAG levels did not exhibit a clinically meaningful correlation with maternal age, as indicated by a low correlation coefficient (Spearman’s r = 0.07145, *p* = 0.0004, Fig. 2A). Further subgroup analyses based on maternal age within each gestational trimester also demonstrated no significant strong correlation (eFigure 1 in Supplementary Materials). In contrast, serum NAG levels showed a robust positive correlation with gestational age (Spearman’s r = 0.6979, *p* < 0.0001, Fig. 2B), clearly indicating a progressive increase in NAG concentrations as pregnancy advanced. We further evaluated the correlations between serum NAG and common physiological parameters (eFigure 2 in Supplementary Materials). A moderate negative correlation was observed between serum NAG and hemoglobin (Spearman’s r = -0.3043, *p* < 0.001), while a weak positive correlation was found with uric acid (r = 0.284, *p* < 0.001). In contrast, no statistically significant correlations were identified between serum NAG and creatinine or urea.

### Differences in serum NAG levels among groups

**Fig. 3:**
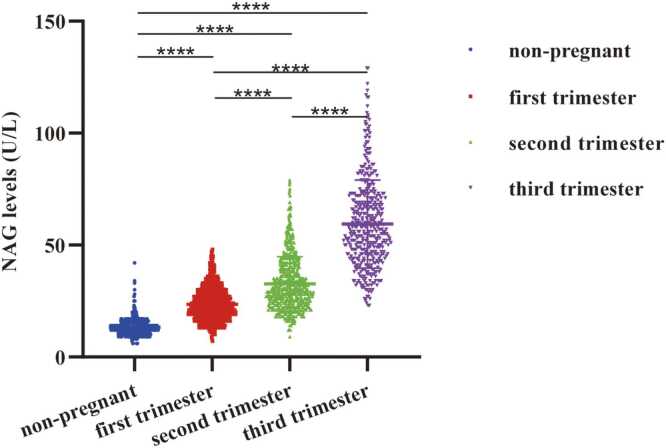
Distribution of NAG levels in non-pregnant women and different pregnancies. first trimester (1–12^+6^ weeks), second trimester (13–27^+6^ weeks), and third trimester (28–40 weeks). When *p* < 0.05 is expressed as *, *p* < 0.01 is expressed as **, *p* < 0.001 is expressed as ***, *p* < 0.0001 is expressed as ****. *Abbreviations*: N-Acetyl-β-D-Glucosaminidase, NAG.

Significant differences in serum NAG levels among the four study groups were identified using the Kruskal-Wallis H test (H=1881.457, p < 0.0001). Subsequent pairwise comparisons adjusted by Bonferroni correction revealed significant differences in serum NAG concentrations between the non-pregnant control group and each of the pregnant subgroups. Moreover, significant differences were also identified among the three pregnancy groups themselves. Serum NAG levels increased gradually and significantly from the first to the third trimester, consistent with the progressive elevation in enzyme activity as pregnancy progressed (Fig. 3).

### Establishment of gestational age-specific reference intervals for serum NAG

Reference intervals for serum NAG concentrations among healthy non-pregnant women and pregnant women across different gestational trimesters were determined using a nonparametric percentile method (P2.5-P97.5), and the results are summarized in Table 2. The reference intervals established were 8.0–23.4 U/L for healthy non-pregnant controls, 12.0–40.0 U/L for the first trimester, 16.0–63.2 U/L for the second trimester, and 29.3–107.0 U/L for the third trimester. Notably, the median serum NAG concentration in late pregnancy (56.0 U/L) represented a 143 % increase compared to early pregnancy (23.0 U/L), underscoring substantial physiological changes in serum NAG during pregnancy progression.

**Table 2:** Reference intervals of N-acetyl-β-D-glucosaminidase (NAG) in healthy non-pregnant women and women at different stages of pregnancy (U/L).

	Non-pregnant group	First-trimester group	Second-trimester group	Third-trimester group
P_2.5_	8.0	12.0	16.0	29.3
P_97.5_	23.4	40.0	63.2	107.0
Median	13.0	23.0	30.0	56.0

## Discussion

This study systematically characterized the gestational trajectory of serum NAG levels in healthy pregnant women. It revealed a significant positive correlation between serum NAG levels and gestational age, accompanied by statistically significant differences across different trimesters. Furthermore, trimester-specific reference intervals were established, filling a critical clinical gap in prenatal biochemical assessment.

Biochemical and histological evidence further supports that serum NAG in pregnancy originates predominantly from placental tissues rather than renal sources. NAG is abundantly expressed in placental decidual and chorionic tissues, which expression profile closely corresponds to histological alterations associated with connective tissue proliferation and placental remodeling [10]. A landmark investigation reported a progressive increase in maternal serum NAG across gestation, followed by a rapid postpartum decline, and identified decidual and chorionic tissues as the primary sites of maximal enzymatic activity [8]. Subsequent placental explant studies confirmed that trophoblasts actively synthesize and secrete β-hexosaminidase/NAG, establishing the placenta as a major physiological contributor to circulating NAG during pregnancy [9]. These findings indicate that serum NAG reflects key processes of placental extracellular matrix remodeling, trophoblastic turnover, and metabolic activity. In our study, urinary NAG was not measured because NAG is a high–molecular-weight enzyme that is not filtered under physiological conditions [2], [3], and all participants were screened to exclude renal or obstetric complications in which urinary NAG would be elevated.

Consistent with previous research [20], [21], we observed a progressive elevation in serum NAG concentrations throughout pregnancy, culminating in a pronounced peak at later gestational stages. Specifically, our data showed a strong correlation between serum NAG and gestational age (Spearman’s p = 0.6979), characterized by a substantial 143 % median increase from early (23.0 U/L) to late pregnancy (56.0 U/L). These data support our interpretation that the gestational increase of serum NAG reflects placental metabolic activity and tissue remodeling rather than renal pathology. Recent placental-secretome mapping has expanded the catalogue of secreted placental proteins that orchestrate maternal adaptations and provide biomarker candidates for placental function and pregnancy outcomes [22]. Within this framework, lysosomal hydrolases measured systemically—such as NAG/HEX—fit the biology of extracellular matrix turnover and syncytiotrophoblast remodeling described in secretome studies, aligning with our observation that serum NAG rises with advancing gestation and may exaggerate under stress states. These insights emphasize the value of trimester-specific reference intervals as a prerequisite for distinguishing physiological elevations from pathological increases in disorders of placental dysfunction.

The appropriateness of using general-population-derived reference intervals for interpreting NAG levels in pregnant women has been previously questioned [16], [17]. Pregnancy induces physiological changes—including increased renal filtration rates—that significantly elevate serum and urinary NAG activity compared to non-pregnant controls. Our study underscores this issue, particularly in the tropical region of Hainan, where high temperature and humidity may further enhance renal hyperfiltration and exacerbate physiological NAG fluctuations during pregnancy. No previous studies have systematically established region-specific, gestational age-dependent reference intervals for serum NAG among pregnant women in this area. This investigation fills this critical void by proposing robust trimester-specific reference ranges: 12.0–40.0 U/L (first trimester), 16.0–63.2 U/L (second trimester), and 29.3–107.0 U/L (third trimester). The significant inter-trimester differences observed likely reflect enhanced NAG-mediated glycosaminoglycan metabolism associated with progressive placental growth and function [18]. Importantly, this study demonstrated no significant strong association of serum NAG levels with maternal age， hemoglobin or other renal function parameters (uric acid, urea, and creatinine)， indicating that they are not a relevant confounder in clinical interpretation.

Notably, the established third-trimester upper reference limit (107.0 U/L) far exceeds the conventional non-pregnancy upper limit (23.4 U/L), clearly demonstrating that current non-pregnancy reference intervals substantially underestimate physiological variations occurring during pregnancy. The rigid application of these non-pregnant reference ranges may thus lead to misclassification of healthy pregnant women as having pathological renal tubular injury, particularly in acute obstetric conditions such as preeclampsia, where NAG concentrations are pathologically elevated [23], [24]. Therefore, the trimester-specific intervals proposed herein offer a practical basis for differentiating physiological increases from true pathological elevations. Although no major professional societies such as ACOG, RCOG, or FIGO currently recommend serum NAG for routine placental assessment, establishing gestational age-specific reference intervals provide an important foundation for future validation studies and potential clinical utility. The clinical diagnostic utility of these intervals, particularly in differentiating conditions such as preeclampsia, warrants further prospective validation.

The strengths of this study include adherence to rigorous methodological standards recommended by the recommendations of the Clinical and Laboratory Standards Institute (CLSI) [25] and the International Federation of Clinical Chemistry and Laboratory Medicine (IFCC) [26]. The total sample size (N = 2416) exceeds the minimum requirement (≥750) by 3.2-fold, as recommended by CLSI for pregnancy reference interval studies. Furthermore, the nonparametric percentile method (P2.5-P97.5) used here appropriately accommodates the non-normal distribution characteristic of serum NAG, offering greater statistical reliability than traditional mean ± 2 SD approaches. Additionally, this investigation represents the first effort to provide clear gestational age-stratified reference intervals, a methodology that more precisely captures the dynamic physiological changes inherent to pregnancy.

However, several limitations must be acknowledged. First, uneven sample distribution across trimesters may introduce selection bias. Second, we did not consider the impact of parity (primiparous versus multiparous) on serum NAG levels; this variable could potentially affect results but remains unaddressed here. Third, this study was limited by the lack of contemporaneous BMI data and detailed dietary records, as these variables were not routinely collected in the retrospective study design. Their potential influence on serum NAG could not be assessed and warrants investigation in future prospective studies. Lastly, it is important to note that this cross-sectional study design restricts our ability to assess intra-individual longitudinal changes in serum NAG throughout pregnancy. Therefore, the reference intervals presented here should be considered as preliminary and population-based. Future prospective, longitudinal studies are essential to confirm these intervals and to evaluate their robustness for tracking changes within individuals over time, which is a prerequisite for confident clinical application.

## Conclusions

In conclusion, this study established robust trimester-specific reference intervals for serum NAG among pregnant women from Hainan and characterized clear gestational age-dependent changes. These data provide an evidence-based g foundation for accurate clinical assessment of maternal renal function and placental status during pregnancy, thereby facilitating improved prenatal care in tropical climates.

## Ethics statement

This retrospective study was approved by the Ethics Committee of the Hainan Women and Children`s Medical Center (protocol code: HNWCMC 2025[77]).

## Funding

This project supported by Hainan Province Clinical Medical Center (QWYH202175).

## CRediT authorship contribution statement

**Yichuan Wang:** Methodology, Investigation, Funding acquisition, Data curation, Conceptualization. **Fen Zhou:** Writing – original draft, Methodology, Funding acquisition, Data curation. **Desheng Wang:** Funding acquisition, Data curation. **Ying Zheng:** Methodology, Funding acquisition, Data curation. **Meng Chang:** Writing – review & editing, Funding acquisition, Conceptualization. **Shichuan Wang:** Writing – review & editing, Methodology, Funding acquisition, Formal analysis.

## Declaration of Competing Interest

The authors declare that the research was conducted in the absence of any commercial or financial relationships that could be construed as potential conflicts of interest.

## Data Availability

The raw data generated and analyzed during the current study are available from the corresponding author upon reasonable request.

## Appendix A Supplementary material

**eFigure 1. Correlation analysis of serum NAG levels with Age in different pregnancies.** First trimester (1–12^+6^ weeks), second trimester (13–27^+6^ weeks), third trimester (28–40 weeks). *Abbreviations*: N-Acetyl-β-D-Glucosaminidase, NAG

**eFigure 2. Correlation of Maternal Serum NAG Levels with Physiological Parameters.***Abbreviations*: N-Acetyl-β-D-Glucosaminidase, NAG

## Footnotes

Appendix A Supplementary data associated with this article can be found in the online version at doi:10.1016/j.eurox.2026.100444.
