# Supplementary figures and images for: Trimester-specific reference intervals for serum N-acetyl-β-D-glucosaminidase in healthy pregnant women in Hainan, China
Source: Eur J Obstet Gynecol Reprod Biol X. 2026 Jan 23;29:100444. doi: 10.1016/j.eurox.2026.100444 (PMC12874144; doi:10.1016/j.eurox.2026.100444)

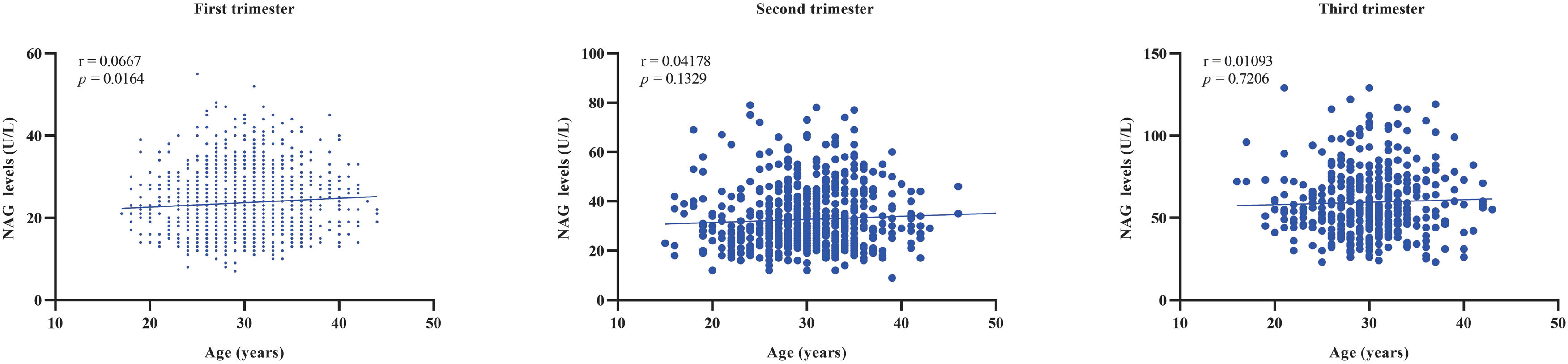

Supplement: Supplementary file 1 — eFigure 1. Correlation analysis of serum NAG levels with Age in different pregnancies. First trimester (1–12+6 weeks), second trimester (13–27+6 weeks), third trimester (28–40 weeks). Abbreviations: N-Acetyl-β-D-Glucosaminidase, NAG [file mmc1.jpg]

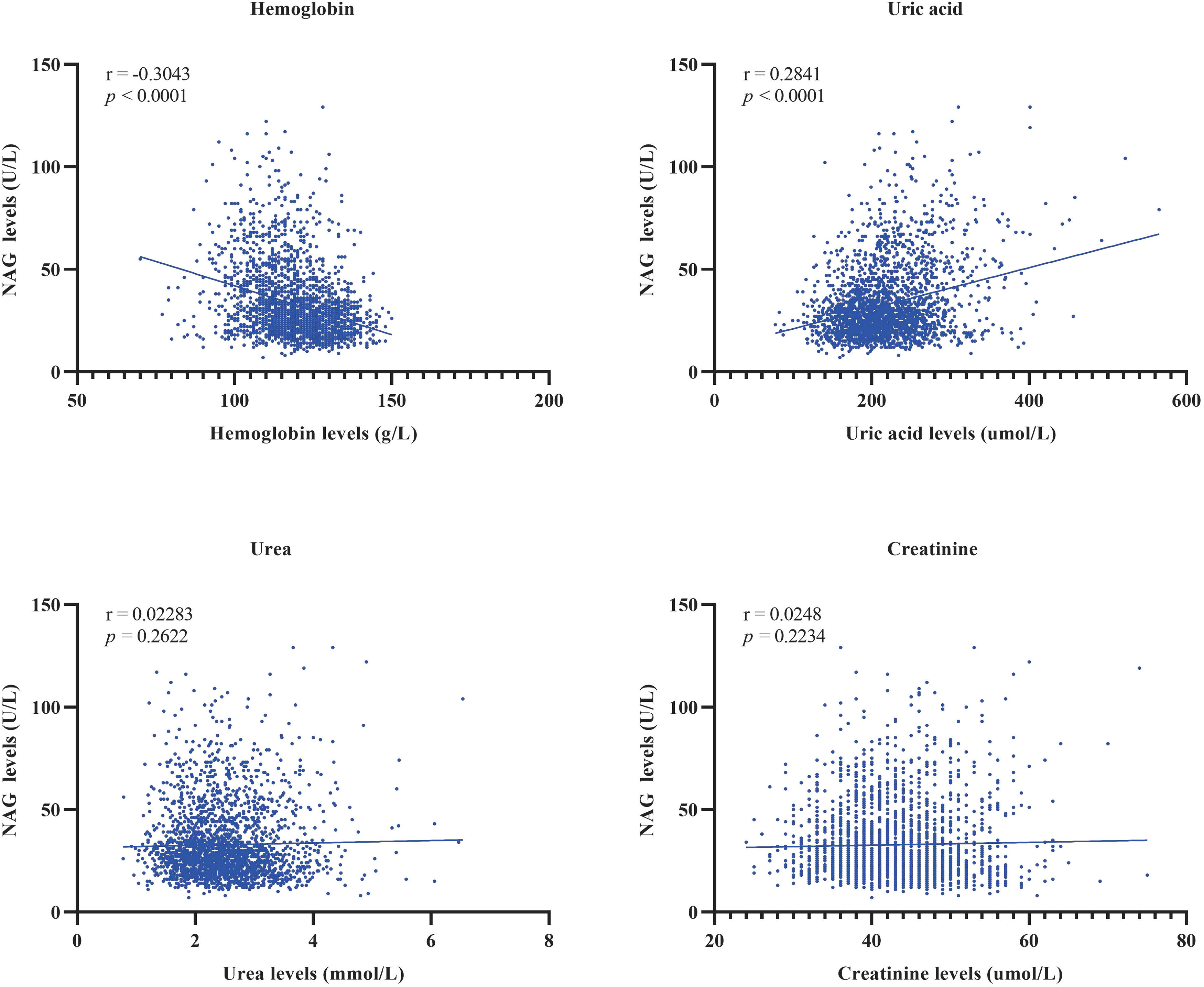

Supplement: Supplementary file 2 — eFigure 2. Correlation of Maternal Serum NAG Levels with Physiological Parameters.Abbreviations: N-Acetyl-β-D-Glucosaminidase, NAG [file mmc2.jpg]
